# A Dive into the Invisible: The Vaginal and Endometrial Microbiota in Gynecologic and Obstetric Disorders: A Narrative Review

**DOI:** 10.3390/life16020344

**Published:** 2026-02-17

**Authors:** Giorgia Schettini, Emilio Pieri, Cristina Rizzo, Matteo Giorgi, Virginia Mancini, Nassir Habib, Ramon Rovira, Gabriele Centini

**Affiliations:** 1 Department of Molecular and Developmental Medicine, Obstetrics and Gynecology, University of Siena, 53100 Siena, Italy; ep.emiliopieri@gmail.com (E.P.); cristina96rizzo@gmail.com (C.R.); 2 Gynecological Unit, Department of Surgical Sciences, Valdarno Hospital, 52025 Montevarchi, Italy; matteogiorgi@hotmail.it; 3 Institute of Pathology, Department of Medical Biotechnology, University Hospital of Siena, 53100 Siena, Italy; virginia.mancini@unisi.it; 4 Department of Obstetrics and Gynecology, Yvette Clinique, 67 Route de Corbeil, 91160 Longjumeau, France; dr.nassirhabib@gmail.com; 5 Department of Gynecologic Oncology, Saint Creu and Saint Pau Hospital, 08025 Barcelona, Spain; ramonroviran@gmail.com; 6 Department of Obstetrics and Gynecology, Alta Val D’Elsa Hospital, Azienda USL Toscana Sud-Est, Campostaggia, 53036 Poggibonsi, Italy

**Keywords:** microbiome, reproductive tract microbiota, vaginal microbiota, endometrial microbiota, gut–uterus axis, estrobolome, dysbiosis, gynecologic diseases, infertility, obstetric outcomes

## Abstract

The human microbiota is increasingly recognized as a key component of women’s reproductive health. This narrative review examines the vaginal, endometrial, and gut microbiota and their roles in the pathogenesis of gynecologic and obstetric disorders, aiming to integrate current evidence into a clinically relevant framework. We review intrinsic (genetic, hormonal, and immunological) and extrinsic (environmental, lifestyle, and pharmacological) factors shaping microbial composition, with particular focus on dysbiosis and the role of the gut estrobolome within the microbiome in estrogen metabolism. The review synthesizes data on microbiota alterations associated with endometriosis, adenomyosis, uterine fibroids, endometrial polyps and hyperplasia, gynecologic malignancies, pelvic inflammatory disease, bacterial vaginosis, infertility, and adverse obstetric outcomes, including preterm birth and fetal growth restriction. Methodological approaches used to characterize the reproductive tract microbiota, such as vaginal swabs, endometrial sampling, and fecal analysis, are critically discussed, together with limitations related to low-biomass environments and contamination risk. Evidence regarding therapeutic modulation of the microbiota, including antibiotics, probiotics, hormonal therapies, and emerging microbiota-based interventions, is summarized, alongside the impact of gynecologic surgery on microbial translocation and long-term microbial balance. Overall, the available literature supports an association between microbiota alterations and multiple reproductive conditions, although causality remains incompletely established. Further standardized and longitudinal studies are needed to clarify mechanisms and guide microbiota-informed diagnostic and therapeutic strategies.

## 1. Introduction

The human *microbiome* encompasses all microorganisms inhabiting the body and their genetic material, while the term *microbiota* refers specifically to these microbial communities within a defined environment [1]. These organisms colonize nearly all mucosal surfaces and play essential roles in homeostasis, immunity, and metabolic balance [2]. Although the gastrointestinal tract hosts the richest and most diverse microbial population, advances in sequencing technologies have identified distinct microbial ecosystems throughout the female reproductive tract, including the vagina, cervix, endometrium, fallopian tubes, ovaries and even the peritoneal cavity, each with unique compositions and functions [3,4].

The gut microbiota, dominated by *Firmicutes*, *Bacteroidetes*, *Actinobacteria*, and *Proteobacteria*, contributes to digestion, vitamin synthesis, immune modulation, and protection against pathogens [1,5]. Its equilibrium is crucial for maintaining intestinal barrier integrity and systemic immune tolerance; disruption of this balance, known as dysbiosis, has been associated with inflammatory and metabolic diseases (inflammatory bowel disease, obesity, metabolic syndrome…), as well as reproductive disorders [3,6].

In contrast, the vaginal microbiota of healthy women is typically dominated by *Lactobacillus* species (*L. crispatus*, *L. jensenii*, *and L. iners*) [7], which maintain a protective low pH environment through lactic acid production [4]. Its composition is dynamic and influenced by hormonal fluctuations, sexual activity, hygiene practices, and environmental factors. Increased microbial diversity, particularly due to anaerobic or facultative anaerobic species, has been linked to impaired vaginal health. Pathogens or dysbiosis-associated taxa such as *Chlamydia trachomatis*, *Gardnerella vaginalis*, *Prevotella* species (spp.), *Sneathia* spp., *Bacteroides* spp., *Mobiluncus* spp., and *Atopobium vaginae* are frequently associated with dysbiosis and adverse gynecological outcomes [2,8], including bacterial vaginosis, pelvic inflammatory disease (PID), infertility, endometriosis, gynecologic malignancies and obstetric complications [3].

Advances in next-generation sequencing (NGS) have challenged the historical assumption of a sterile upper genital tract. Low-biomass microbial communities have been detected in the uterus, fallopian tubes, and ovaries, forming a continuum with the lower tract [9]. This upper-tract microbiota, generally more diverse but less abundant than the vaginal one, includes *Lactobacillus*, *Pseudomonas*, *Propionibacterium*, *Acinetobacter*, *and Streptococcus* [2]. Although its functional role remains incompletely defined, it may contribute to immune modulation, tubal function, fertility, and inflammatory gynecological conditions.

The endometrial microbiota, despite its low microbial load, appears clinically relevant. Predominant genera include *Lactobacillus*, *Bifidobacterium*, *and Streptococcus*, and have been associated with implantation success, pregnancy maintenance, and susceptibility to endometrial pathology. Similarly, the cervical microbiota acts as an ecological and immunological interface between the vagina and uterus, shaping infection risk and cervical carcinogenesis [3,4].

The peritoneal cavity, once considered sterile, may harbor microbial DNA and viable bacteria, potentially originating from the genital tract or intestinal translocation. These microorganisms have been associated with inflammatory processes underlying conditions such as endometriosis or PID, although their pathogenic role remains debated [4,6].

Overall, interactions between site-specific microbial ecosystems and host immune-endocrine networks are fundamental to women’s reproductive health. Microbial imbalance at any anatomical site may alter immune response, hormonal signaling, and tissue homeostasis, contributing to gynecological and obstetric disorders. Understanding the composition and dynamics of the microbiota across the gastrointestinal and reproductive tracts is therefore essential for advancing diagnostic approaches and developing rational, microbiota-targeted therapeutic strategies [10].

## 2. Materials and Methods

This narrative review aimed to provide an updated synthesis of current evidence on the relationship between microbiota composition, dysbiosis, and major gynecological and obstetric diseases. A structured literature search was performed in PubMed/MEDLINE and Google Scholar for studies published between January 2010 and September 2025, using the keywords “microbiota”, “microbiome”, “gut microbiota”, “vaginal microbiota”, “endometrial microbiota”, “reproductive microbiota”, and “dysbiosis”.

Eligible publications included original research articles, systematic reviews, meta-analyses, and clinical studies in English investigating microbial composition or alterations of the intestinal, vaginal, cervical, endometrial, or peritoneal microbiota in women. Studies based exclusively on animal models or lacking microbiological or molecular analyses were excluded. Titles and abstracts were independently screened by three reviewers (G.S., G.C., and E.P.), and full texts meeting inclusion criteria were assessed for methodological quality, clinical relevance, and consistency of microbiota profiling techniques.

Priority was given to human studies employing molecular approaches (16S rRNA sequencing, shotgun metagenomics, or quantitative PCR), with clearly defined patient populations and clinically relevant outcomes. Data were synthesized narratively, emphasizing proposed pathophysiological mechanisms linking dysbiosis to inflammation, hormonal regulation, immune activation, and reproductive dysfunction.

As this was a narrative review, no formal quality scoring system (e.g., PRISMA) or quantitative meta-analysis was applied. However, heterogeneity in study design, population characteristics, sampling techniques, and sequencing methodologies (particularly in low-biomass environments) was carefully considered during interpretation to avoid overstatement of causal relationships.

### Strength of Evidence

Most data in reproductive microbiota research derive from observational cohort and cross-sectional studies, whereas randomized controlled trials (RCTs) and longitudinal interventional studies remain limited. Systematic reviews and meta-analyses provide the highest level of evidence, particularly in bacterial vaginosis, preterm birth, and gynecologic oncology. In contrast, findings from low-biomass uterine or peritoneal samples required cautious interpretation, as reported microbiota alterations largely reflect associations rather than causal relationships. Microbiota analyses of the endometrium and upper genital tract are intrinsically challenged by low-biomass samples, which increases susceptibility to contamination and technical variability. Together with heterogeneous sequencing approaches and small sample sizes, these limitations restrict causal inference and partly explain inconsistencies across studies.

From a clinical perspective, microbiota assessment remains primarily investigational; however, identification of *Lactobacillus* depletion or bacterial vaginosis (BV)-like profiles may support risk stratification and adjunctive management in selected patients, particularly in ART settings or recurrent disease.

## 3. Etiopathogenesis of the Normal vs. Altered Microbiota

The composition of a healthy microbiota reflects a dynamic balance shaped by intrinsic (age, genetics, ethnicity, immune reactivity and hormonal milieu) and extrinsic factors (diet, physical activity, stress, smoking, sexual behavior, hygiene habits, and exposure to endocrine-disrupting chemicals), all of which influence microbial diversity and function along the gut-reproductive axis [11,12]. These determinants regulate the equilibrium between protective taxa, such as *Lactobacillus* spp. in the reproductive tract and short-chain fatty acid (SCFA)-producing bacteria in the gut, and potentially pathogenic organisms that promote inflammation or epithelial dysfunction [13].

Microbial colonization is highly dynamic over time. Longitudinal studies show that individual microbiota profiles fluctuate with physiological transitions, lifestyle changes, pregnancy or medical interventions [14]. Antibiotics, hormonal therapies, chemotherapy and surgical procedures can significantly reshape microbial ecosystems, sometimes resulting in persistent dysbiosis [15,16]. Birth-related factors are also crucial: vaginal birth exposes newborns to maternal vaginal and intestinal flora, rich in *Lactobacillus* and *Bifidobacterium*, whereas cesarean-section favors early colonization by skin-associated taxa such as *Staphylococcus* and *Corynebacterium*, with downstream effects on immune and metabolic development [17].

Diet represents one of the strongest modulators of the microbiota. Fiber-rich diets enhance microbial diversity, SCFA production, gut barrier integrity and immune tolerance, while low-fiber or high-sugar diets reduce microbial resilience and favor pro-inflammatory taxa [5]. When intrinsic and extrinsic stressors exceed host adaptive capacity, the system may shift toward a low-diversity, dysbiotic state characterized by anaerobic or opportunistic overgrowth, epithelial barrier disruption and increased susceptibility to inflammation and infection.

Among nutritional factors, vitamin D plays a key role in microbiota homeostasis. Vitamin D receptors (VDRs), expressed in the gut, immune system, liver, and uterus [18], regulate over 1000 genes involved in microbial balance, gut barrier integrity, immune modulation and pathogen defense [19]. Adequate vitamin D levels support gut microbiota diversity, promoting beneficial taxa such as *Bacteroidetes*, *Verrucomicrobia*, and *Actinobacteria*, including *Akkermansia muciniphila*, which contribute to mucosal homeostasis and immune tolerance [20]. Conversely, vitamin D deficiency is linked to a reduced *A. muciniphila*, increased *Firmicutes*, greater gut permeability and systemic inflammation, contributing to dysbiosis [21]. Vitamin D/VDR signaling also suppresses bacteria-induced NF-κB activation and pro-inflammatory cytokine (TNF-α, IL-1β, IL-6) [22,23], while supplementation boosts anti-inflammatory mediators (IL-4, IL-10) and partially restores microbial balance [22]. Overall, vitamin D emerges as a modulator of both gut and reproductive tract microbiota, with potential implications for immune homeostasis and gynecological health [22,24].

## 4. Methods for Investigating the Female Genital and Gut Microbiota

Investigating the vaginal and endometrial microbiota requires highly standardized sampling and analytical protocols, as the low biomass of the endometrium and the risk of cervicovaginal contamination necessitate careful methodological control [4,25]. Endometrial microbial composition varies with inflammatory status, hormonal fluctuations and pregnancy; therefore, sampling procedures must explicitly address contamination risk during cervical passage. For vaginal microbiota assessment, self-collected swabs offer a reliable, convenient, and cost-effective option. Forney et al. reported no significant differences between self- and clinician-collected samples [26].

Early studies relied on aspirated endometrial fluid obtained via catheters or Pipelle devices; however, current evidence supports endometrial biopsy as more representative of microbial diversity [27]. To minimize transcervical contamination, techniques employing protective sheaths, such as the Cornier Pipelle described by Liu et al. (2018), have been adopted [28]. Sampling time is also essential due to cyclical microbial variability along the menstrual cycle [29]. The mid-follicular phase is generally preferred to limit hormonal confounding [30], while recent antibiotic use or active bleeding constitute exclusion criteria [31].

Over the past 15 years, microbiota characterization has evolved from subjective microscopic evaluation to molecular techniques, such as quantitative PCR and NGS [32]. 16S rRNA gene sequencing enabled classification of vaginal microbiota into *community state types* (CSTs):

- CST I (*L. crispatus* dominance);
- CST II (*L. gasseri* dominance);
- CST III (*L. iners* dominance);
- CST IV (heterogeneous communities with low *Lactobacillus* and high *anaerobes*);
- CST V (*L. jensenii* dominance).

While *Lactobacillus*-dominated CSTs reflect a protective state, CST IV is associated with bacterial vaginosis and increased susceptibility tosexually transmitted infections (STIs) [33]. For species-level resolution and functional profiling, shotgun metagenomics is increasingly favored [34].

Despite technological advances, heterogeneity in 16S rRNA primer selection remains a limitation, contributing to inter-study variability. Standardization of sampling, sequencing, and bioinformatic pipelines remains a key unmet need [28]. Best practices include immediate sample preservation (e.g., at −80 °C), host DNA depletion, and optimized microbial lysis to ensure accurate representation of low-biomass genital microbiota [31].

## 5. Microbiota and Gynecological Disorders

### 5.1. Microbiota, Endometriosis and Adenomyosis

Endometriosis and adenomyosis are chronic estrogen-dependent disorders marked by ectopic or infiltrating endometrial-like tissue, persistent inflammation, hormonal imbalance and immune dysregulation. Growing evidence indicates that microbial dysbiosis may modulate these processes and contribute to disease onset and progression [35].

#### 5.1.1. Microbiota and Endometriosis

Women with endometriosis typically show reduced protective *Lactobacillus* spp. in the vaginal microbiota and increased opportunistic taxa such as *Gardnerella vaginalis*, *Atopobium vaginae* and *Ureaplasma* spp. [32]. Endometrial samples reveal a shift toward pro-inflammatory communities, with increased *Fusobacterium*, *Streptococcus*, *Enterococcus*, *Escherichia coli* and *Prevotella* and decreased *Lactobacillus* spp. [36].

Gut dysbiosis presents in endometriosis, can impair the intestinal barrier, leading to increased permeability (“leaky gut”) and translocation of bacterial products such as lipopolysaccharides (LPS) into the circulation, triggering systemic and pelvic inflammation [37].

The estrobolome also plays a role: β-glucuronidase-producing bacteria (*E. coli*, *Bacteroides fragilis*, *Streptococcus agalactiae*) increase estrogen deconjugation and reabsorption, raising circulating estrogen levels and sustaining endometrial tissue proliferation and ectopic lesion growth [38].

The bacterial contamination theory further links dysbiosis to endometriosis: retrograde menstruation may introduce endotoxin-rich (such as LPS) endometrial cells into the peritoneal cavity, especially when vaginal or gut dysbiosis increases Gram-negative bacteria. This activates TLR4 signaling, NF-κB and COX-2 pathways, stimulating angiogenesis, fibrosis and survival of lesions [32,38,39]. Peritoneal fluid from affected women shows enrichment of *Prevotella*, *Atopobium*, *Veillonellaceae* and *Comamonas*, suggesting ascending or translocated bacteria participate in pathogenesis [6].

#### 5.1.2. Microbiota and Adenomyosis

Although data are more limited, adenomyosis also shows altered reproductive-tract microbiota, with decreased *Lactobacillus* dominance and increased *Gardnerella* and *Prevotella* spp. in uterus and cervix, as well as greater vaginal richness and shifts involving taxa such as *Oscillospirales* and *Ruminococcaceae* [40]. These changes may disrupt the local myometrial immune environment, promoting cytokine release, tissue remodeling and endometrial glandular infiltration. Estrobolome-related estrogen reactivation observed in endometriosis may similarly contribute to the hyperestrogenic milieu of adenomyosis [35].

Given shared features (chronic inflammation, immune activation, estrogen dependence, tissue invasion) and overlapping dysbiosis patterns, endometriosis and adenomyosis may involve common microbiota-mediated inflammatory mechanisms.

Collectively, dysbiosis may trigger a cascade involving immune activation, increased gut permeability, endotoxin translocation, altered estrogen metabolism and direct microbial contamination of the peritoneal cavity, sustaining chronic inflammation and hormonal imbalance [41].

Although causality is unconfirmed and human data remain heterogeneous, microbiome-targeted strategies (probiotics, prebiotics or dietary interventions) are emerging as promising adjuncts for estrogen-driven gynecological diseases [40,41].

### 5.2. Microbiota and Fibromatosis

Recent evidence supports a gut–uterus axis in the onset and progression of uterine fibroids (UFs), with dysbiosis emerging as a contributor to hormonal, metabolic and inflammatory alterations that favor fibroid growth [42]. Women with UFs often show reduced gut microbial diversity, enrichment of pro-inflammatory or estrogen-modulating taxa such as *Bacteroides*, *Prevotella*, *Ruminococcus*, and β-glucuronidase-producing *Enterobacteriaceae* [43,44] as well as reduced production of SCFAs, key regulators of immune balance and smooth-muscle proliferation [43,44]. These changes promote enhanced estrogen recycling via the estrobolome, contributing to the hyperestrogenic milieu typical of fibroid development [45].

At the genital-tract level, fibroid patients display increased anaerobic species linked to inflammation and extracellular matrix accumulation, including *Gardnerella vaginalis*, *Atopobium vaginae*, *Sneathia sanguinegens*, and *Prevotella bivia*, alongside reduced *L. crispatus* and *L. jensenii* [46]. Such alterations may influence fibroid biology through toll-like receptor (TLR) activation, oxidative stress and dysregulated cytokines (IL-6, TNF-α and TGF-β), all implicated in leiomyoma pathophysiology [47].

Interestingly, fibroid tissue itself may harbor distinct microbial DNA signatures. Higher prevalence of *Acinetobacter*, *Pseudomonas*, *Streptococcus*, and *Cutibacterium acnes* has been identified within leiomyomas compared with adjacent myometrium, suggesting a local microbial niche that may affect tumor behavior [47].

Microbiota composition may also modulate treatment response. For example, vitamin D supplementation appears more effective in restoring endometrial immune balance when supported by a favorable gut microbial profile, indicating a potential synergy between microbiome-targeted strategies and medical therapy [24].

Overall, emerging data depict uterine fibroids not solely as hormone-dependent lesions but as a condition embedded within a broader microbial–immune–endocrine network, in which gut and genital microbiota may influence susceptibility, progression, and therapeutic outcomes [46].

### 5.3. Microbiota and Endometrial Polyps

Growing evidence indicates that women with endometrial polyps (EPs) exhibit distinct alterations in both vaginal and intrauterine microbiota. These changes consistently reflect a shift away from Lactobacillus dominance toward an over-representation of pro-inflammatory or potentially pathogenic taxa.

Tian et al. reported significantly reduced vaginal *Lactobacillus* spp., particularly *L. crispatus*, in women with endometrial polypoid lesions, accompanied by enrichment of *Gardnerella*, *Prevotella*, *Atopobium* and *Sneathia*, a pattern resembling bacterial vaginosis-like dysbiosis and associated with local inflammation [48]. Similarly, Zhao et al., using 16S rRNA sequencing of endometrial samples, reported a higher microbial diversity and increased *Proteobacteria, Bacteroidetes*, *Escherichia*/*Shigella*, *Streptococcus*, and *Enterococcus* in EP patients, suggesting that a dysbiosis may contribute to epithelial proliferation and immune dysregulation [49].

Vanakova et al. further confirmed that the intrauterine microbiota in EPs is characterized by reduced Lactobacillus prevalence and increased opportunistic genera such as *Gardnerella*, *Prevotella*, and *Peptoniphilus* [50], changes linked to heightened inflammatory signaling and impaired mucosal immunity.

Collectively, these findings support a reproducible dysbiosis pattern in EPs, marked by lower Lactobacillus and higher abundance of anaerobic and facultative anaerobic bacteria. Whether dysbiosis is a cause, consequence, or co-factor in polyp development remains unclear, but these microbiota signatures may hold diagnostic or therapeutic relevance [48].

Across gynecological disorders, available evidence linking microbiota alterations to disease pathogenesis is largely derived from small case–control or cross-sectional studies, with heterogeneous diagnostic criteria, sampling sites, and analytical pipelines. Cohort sizes are frequently limited, and important confounders (such as hormonal treatments, body mass index, diet, sexual behavior, and prior antibiotic exposure) are often incompletely controlled. As a result, reported microbial signatures should be interpreted as associative rather than causal, and inter-study variability remains substantial. To date, no longitudinal or interventional human studies have conclusively demonstrated a causal role of microbiota dysbiosis in the onset or progression of estrogen-dependent gynecological disorders. Nevertheless, the recurrent observation of shared dysbiotic patterns across conditions suggests that microbiota alterations may act as disease modifiers or co-factors rather than primary drivers, supporting cautious exploration of microbiota-targeted strategies as adjunctive, rather than standalone, therapeutic approaches.

### 5.4. Microbiota, Sexually Transmitted Infections and Pelvic Inflammatory Disease

Epidemiological evidence consistently indicates that a *Lactobacillus*-dominated vaginal microbiota is associated with reduced susceptibility to sexually transmitted infections (STIs). A meta-analysis by Tamarelle et al. demonstrated that high-*Lactobacillus* community states significantly lower the risk of *Human Papillomavirus* (HPV) and *Chlamydia trachomatis* acquisition [51]. Experimental data further support this association: Nardini et al. showed that *L. crispatus* supernatants rich in lactic acid directly inhibit *C. trachomatis* infectivity in vitro [52]. Collectively, these findings suggest that a low-diversity, *Lactobacillus*-rich microbiota supports epithelial barrier integrity and creates an unfavorable environment for pathogens.

Conversely, vaginal dysbiosis, especially BV, is a well-established risk factor for multiple STIs. Women with BV exhibit significantly higher rates of *Chlamydia*, *Neisseria gonorrhoeae*, *HIV*, and other infections [53]. Molecular profiling confirms severe depletion of *L. crispatus* and increased anaerobes such as *Gardnerella* and *Prevotella* in STI-positive women [54,55]. These anaerobes secrete sialidases, proteases and SCFA that disrupt cervical mucus and epithelium, increase vaginal pH, and promote local inflammation, thereby facilitating pathogen persistence and ascension [54,56].

Behavioral factors further modulate risk. High-risk populations, including sex workers and adolescents, display more diverse, *Lactobacillus*-poor vaginal microbiota and higher STI incidence. Wessels et al. reported reduced *Lactobacillus* abundance and increased microbial diversity in sex workers [57], while Mehta et al. found that adolescents who subsequently acquired STIs had lower baseline *L. crispatus* levels [58].

The same microbiological principles extend to PID, which represents the most severe clinical outcome of ascending genital tract infection. A stable *Lactobacillus*-dominated vaginal microbiota limits inflammation and microbial ascension, whereas persistent dysbiosis favors overgrowth of BV-associated anaerobes that degrade the cervical barrier [54,56].

Beyond classical sexually transmitted pathogens, increasing evidence implicates BV-associated communities in PID pathogenesis. Depletion of protective *Lactobacillus* spp. and enrichment of *Gardnerella vaginalis*, *Atopobium vaginae*, *Prevotella*, *Sneathia*, and *Mobiluncus* facilitate bacterial ascent into the uterus and fallopian tubes [51,54,55]. These taxa activate toll-like receptor-mediated inflammatory pathways, increasing IL-6, IL-8, and TNF-α production and promoting tubal inflammation and fibrosis [56]. Molecular studies consistently show a shared dysbiotic signature between BV and PID, characterized by profound *L. crispatus* depletion, with enrichment of anaerobic genera [54,55,56]. Clinically, this inflammatory milieu contributes to long-term sequelae including chronic pelvic pain, ectopic pregnancy, and infertility. However, most available data are observational, and causality remains unproven [51,52,53,54,55,56,57,58].

### 5.5. Microbiota and Vaginosis

BV occurs when the normal *Lactobacillus*-dominated vaginal ecosystem is replaced by a diverse anaerobe-rich community. This transition involves a marked depletion of *Lactobacilli* and overgrowth of facultative and strict anaerobes, such as *Gardnerella vaginalis*, *Atopobium vaginae*, *Prevotella* species, *Mobiluncus* species, *Sneathia*/*Leptotrichia*, *Peptostreptococcus and Mycoplasma* [8]. Reduced lactic acid production results in elevated vaginal pH (>4.5). Importantly, BV does not reflect infection by a single pathogen but rather a polymicrobial ecological imbalance, in which different bacterial assemblages can produce a similar clinical phenotype [8].

Molecular studies have identified three distinct bacterial vaginosis–associated bacteria, termed BVAB1, BVAB2, and BVAB3, strongly linked to BV:

- BVAB1, now classified as *Candidatus Lachnocurva vaginae*, is a fastidious anaerobe enriched in BV and closely associated with polymicrobial biofilm formation together with *Gardnerella vaginalis*;
- BVAB2 is phylogenetically related to the anaerobic *Firmicutes* (family *Ruminococcaceae*);
- BVAB3 also belongs to the *Firmicutes* phylum and is distinct from both *Gardnerella* and *Atopobium* [59].

BVAB1-3 are rarely detected in *Lactobacillus*-dominated eubiotic communities but are consistently enriched in BV and associated with vaginal inflammation, persistence and recurrence of BV after antibiotic treatment, and adverse reproductive outcomes, including preterm birth [59,60].

A defining featureof BV is polymicrobial biofilm formation on the vaginal epithelium. *G. vaginalis* plays a central role by adhering to epithelial cells and co-aggregating with other BV-associated taxa, particularly *Atopobium vaginae* [60]. Biofilm-associated bacteria are shielded from antibiotics and host immune responses, contributing to high recurrence rates, exceeding 50% within 12 months after standard therapy [61].

Recurrence is often associated with persistence of BV-associated taxa following treatment. Women who relapse show higher post-treatment abundance of *A. vaginae*, *Gardnerella* or *Aerococcus* compared with women achieving long-term remission [62]. Computational modeling further suggests that specific baseline community states predispose to persistence and recurrence [63].

A major limitation of antibiotic therapy is its inability to restore protective *Lactobacillus* dominance. After treatment, the vaginal niche may remain insufficiently colonized by *Lactobacilli*, allowing BV-associated bacteria to rebound, especially if protected in biofilms or reintroduced by partners. This has driven interest in adjunctive microbiota-directed interventions [64]. A randomized placebo-controlled trial by Cohen et al. demonstrated that intravaginal *L. crispatus* (LACTIN-V) administered after Metronidazole significantly reduced BV recurrence at 12 weeks by 34% and achieved sustained colonization of the administered strain [65]. Among gynecological disorders, BV is one of the few conditions supported by randomized clinical trial data for microbiota-based interventions; nevertheless, recurrence remains common, underscoring the multifactorial nature of vaginal dysbiosis.

### 5.6. Microbiota and Cancer

#### 5.6.1. Microbial Dysbiosis and Oncobiosis

Advances in 16S rRNA sequencing show that dysbiosis is a recurrent feature of gynecological malignancies. When associated with cancer, this altered microbial state is commonly referred to as “*oncobiosis*” and may represent a modifiable disease-associated signature [66]. Evidence in gynecologic oncology is largely derived from systematic reviews and meta-analyses. Vizza et al. analyzed 21 studies and identified reproducible oncobiotic patterns across gynecological cancers, with microbial profiles correlating with tumor type, grade, and histology [3]. Nevertheless, most included studies were retrospective and heterogeneous in sampling and sequencing methodologies, and mechanistic insights are primarily derived from small exploratory cohorts.

#### 5.6.2. Endometrial Cancer

Endometrial cancer (EC) exhibits a characteristic oncobiotic signature. Concurrent detection of *Atopobium vaginae* and *Porphyromonas somerae*, particularly in the context of elevated vaginal pH, demonstrated high diagnostic accuracy in a single case–control cohort by Walther-António et al. (73–93% sensitivity and 67–90% specificity) [67]. Although sensitivity and specificity were promising, external validation in independent populations is lacking. Transcriptomic analyses have identified over 5500 metabolically active bacterial species in EC, with pathway enrichment linked to epithelial–mesenchymal transition and tumor invasion [68]. Vaginal community state types also appear to cluster by tumor grade, with *Lactobacillus*-dominated profiles in benign disease (*L. crispatu*) and anaerobe-rich communities (*L. iners*, *Fusobacterium ulcerans* and *Prevotella bivia*) in high-grade EC [69]. CST-based stratification of tumor grade was derived from moderate-sized observational cohorts and has not yet been confirmed in randomized or prospective screening studies.

Mechanistic studies suggest that specific taxa (e.g., *Anaerococcus vaginalis*) may contribute to inflammatory and metabolic tumor-promoting pathways, including reactive oxygen species generation [70], estrogen reactivation via β-glucuronidase activity (because of fungal dysbiosis, particularly of *Penicillium*) [71], and angiogenesis [3] through succinate signaling [3,70,71]. Despite consistent associative patterns, no longitudinal or interventional studies currently demonstrate a causal role or support clinical implementation of microbiota-based screening tools.

#### 5.6.3. Ovarian Cancer

Ovarian cancer exhibits compartment-specific oncobiosis. Vaginal microbiota, especially in BRCA1/2 mutation carriers, shows marked *Lactobacillus* depletion [72]. Upper genital tract and peritoneal samples are enriched in Gram-negative taxa (*Acinetobacter*, *Sphingomonas and Methylobacterium*), with reduced microbial diversity compared to normal fallopian tube tissue [73], features that may facilitate metastatic spread and inflammatory signaling [74]. Lysophosphatidic acid accumulation in ascites, derived from Gram-negative lysophospholipids, further promotes proliferation and invasion [72]. Sexual transmission of *Chlamydia trachomatis* increases ovarian cancer risk by 90-fold in high-antibody-titer women, establishing infection-dysbiosis synergy in ovarian carcinogenesis [72].

#### 5.6.4. Cervical Cancer

Although HPV is the primary etiological driver of cervical cancer, vaginal dysbiosis strongly influences HPV acquisition, persistence, and malignant progression [66]. *Lactobacillus* depletion and enrichment of BV-associated taxa (*Gardnerella vaginalis*, *anaerobes*) increase HPV persistence and delay viral clearance [66]. Dysbiotic microbiota promote cervical inflammation via TLR signaling and cytokine-mediated pathways (IL-6/IL-8), whereas *Lactobacillus* species support epithelial integrity, IgA production, and immune tolerance [72].

Overall, oncobiosis represents a shared feature across gynecological cancers, with microbial signatures linked to disease phenotype and treatment response. While the modifiable nature of the microbiota makes it an attractive target for biomarker development and adjunctive interventions, current evidence is largely associative. Given the low-biomass nature of upper genital tract samples and methodological variability, large prospective studies are required before microbiota-based strategies can be translated into clinical practice.

### 5.7. Microbiota and Infertility

Infertility affects up to 15% of couples worldwide, and a substantial proportion of cases remain idiopathic despite advances in assisted reproductive technologies (ARTs). Increasing evidence suggests that alterations of the genital tract microbiota may contribute to infertility by disrupting immune homeostasis, impairing gamete function and compromising endometrial receptivity [75,76].

In healthy women, the vaginal microbiota is typically dominated by *Lactobacillus* spp. (*L. crispatus*, *L. jensenii*, *L. gasseri*), which maintain a low pH and protect against pathogenic colonization [77]. Vaginal dysbiosis, characterized by *Lactobacillus* depletion and enrichment of *Gardnerella vaginalis*, *Atopobium vaginae*, or *Prevotella* spp., has been associated with lower implantation and pregnancy rates, especially in in vitro fertilization (IVF) settings [75,78]. Elevated levels of Gram-negative lipopolysaccharide have been detected in menstrual effluents of women with implantation failure, supporting a link between microbial components and local inflammatory activation [78].

The uterine cavity, previously considered sterile, is now recognized as a low-biomass microbial environment contiguous with the lower genital tract [4]. NGS-based studies have identified microbial communities in the endometrium and follicular fluid, suggesting functional communication between reproductive compartments [79]. A *Lactobacillus*-dominant endometrial microbiota has been correlated with higher implantation and live-birth rates, whereas increased diversity or enrichment of taxa such as *Streptococcus*, *Prevotella* or *Gardnerella* correlates with poorer IVF outcomes, likely through inflammation-mediated impairment of endometrial receptivity [79,80].

Emerging data also highlights the contribution of the male genital microbiota to couple fertility. Semen harbors a diverse microbial community and enrichment of genera such as *Prevotella*, *Finegoldia*, and *Campylobacter* spp. has been linked to reduced sperm motility, abnormal morphology, and increased DNA fragmentation [81]. Microbial exchange between sexual partners may further influence vaginal *Lactobacillus* dominance and contribute to unexplained infertility [82]. Metagenomic studies in infertile couples undergoing ART reveal significant microbial overlap between partners [80], with male genital samples often enriched in BV-associated-taxa [79,80]. In a pivotal study, Baud et al. (2019) demonstrated that male genital microbiota frequently includes *Prevotella*, *Finegoldia*, *Porphyromonas*, and *Mobiluncus*, supporting the hypothesis that the male partner may act as a microbial reservoir influencing the vaginal microbiota composition and female reproductive outcomes [83].

Therapeutic manipulation of the reproductive microbiota remains experimental. Empirical antibiotic use before IVF has shown inconsistent benefits, and probiotic supplementation has yielded inconclusive results [84,85]. At present, associations between microbiota composition and fertility outcomes are largely derived from observational ART cohorts, and randomized trials demonstrating improved reproductive outcomes following microbiota modulation remain limited. Robust longitudinal and interventional studies are required to establish causality and guide personalized microbiota-based strategies [86].

### 5.8. Microbiota and Obstetric Complications

The maternal microbiota plays a pivotal role in pregnancy, influencing maternal health, fetal development and neonatal microbial colonization. Vaginal, cervical, placental, or intestinal dysbiosis has been increasingly associated with obstetric complications, including preterm birth, preeclampsia, gestational diabetes mellitus (GDM), and premature rupture of membranes (PROM) [76,86]. During healthy pregnancy, the vaginal microbiota is typically dominated by *Lactobacillus* spp. (*L. crispatus*, *L. jensenii*), which maintain an acidic environment (pH < 4.5) through lactic acid and bacteriocin production. Reduced *Lactobacillus* abundance with enrichment of anaerobes (*Gardnerella vaginalis*, *Atopobium vaginae*, *Prevotella*, and *Ureaplasma*) is strongly correlated with BV and adverse pregnancy outcomes, particularly spontaneous preterm birth and chorioamnionitis [87].

Beyond the lower genital tract, the existence and role of a distinct placental or intrauterine microbiota remain debated [88]. Nevertheless, dysbiotic microbial signals, arising from ascending infection or hematogenous spread from the oral cavity or gut, may contribute to sterile intrauterine inflammation and adverse outcomes, including preterm labor, fetal growth restriction, and early pregnancy loss [87,88,89]. Women with recurrent miscarriage often display reduced *Lactobacillus* dominance and increased colonization by *Gardnerella*, *Atopobium*, and *Streptococcus* spp., implicating altered vaginal and endometrial microbiota in impaired implantation and placentation [89].

Maternal dysbiosis composition also influences neonatal health. Delivery mode critically shapes early microbial colonization: vaginal delivery promotes tranfer of *Lactobacillus-*, *Bacteroides-* and *Bifidobacterium-rich microbiota*, whereas elective cesarean section is associated with delayed microbial maturation and enrichment of skin-associated taxa (*Staphylococcus* and *Corynebacterium*) [90]. Cesarean delivery after labor onset partially restores microbial exposure and results in neonatal profiles closer to those observed after vaginal birth [5,91].

Meta-analyses provide strong evidence linking vaginal dysbiosis and anaerobe-dominated communities with increased risk of spontaneous preterm birth and chorioamnionitis [86,87]. Proposed mechanisms include ascending infection, inflammatory activation of fetal membranes, and metabolite-mediated sterile inflammation [87,88,89,90,91]. While causality remains under investigation, these findings support the potential role of genital tract microbiota composition in preterm birth risk stratification. Importantly, evidence linking bacterial vaginosis and anaerobe-dominated vaginal microbiota to preterm birth is supported by high-level meta-analytic data, whereas findings regarding placental or intrauterine microbiota are largely derived from low-biomass sequencing studies and remain methodologically controversial.

## 6. Uterine–Gut Microbiota Axis and Its Implications for Gynecological Health

Growing evidence supports a bidirectional interaction between intestinal and uterine microbiota, commonly referred to as the gut–uterus axis [4,9]. Through effects on systemic immunity, metabolism and estrogen homeostasis, the gut microbiota may influence endometrial microbial composition and immune tone. In dysbiotic states, increased intestinal permeability (“leaky gut”) facilitates translocation of bacterial metabolites and endotoxins, such as LPS, into the bloodstream, promoting chronic low-grade inflammation and immune activation [6].

Microbial and immunological crosstalk between the gut and reproductive tract suggests that intestinal dysbiosis may predispose to vaginal and uterine microbial imbalance [24]. Reduced *Lactobacillus* and *Bifidobacterium* and enrichment of *Proteobacteria* and *Firmicutes* increase β-glucuronidase activity, enhancing estrogen deconjugation and reabsorption and potentially sustaining estrogen-dependent disorders such as endometriosis and adenomyosis [38].

Clinically, a strong overlap exists between gastrointestinal and gynecological conditions. Up to 60–70% of women with endometriosis report irritable bowel syndrome (IBS)-like symptoms, while inflammatory bowel disease (IBD) is associated with increased risk of endometriosis and chronic pelvic pain [92]. Endometriosis is associated with higher prevalence of gastrointestinal disorders, including IBS (odds ratio 2.5–3.0) and functional constipation or diarrhea, indicating shared pathogenic mechanisms involving gut barrier dysfunction, dysbiosis and systemic inflammation [93].

Overall, the gut-reproductive microbiota axis represents a key regulator of women’s health, whereby intestinal homeostasis supports uterine microbial balance and reproductive function, while dysbiosis and increased gut permeability may propagate inflammatory signals that affect the reproductive tract.

## 7. Therapeutic Modulation of the Gut-Reproductive Microbiota

The intestinal and genital microbiota are highly dynamic ecosystems influenced by pharmacological interventions. Antibiotics are among the strongest disruptors of microbial homeostasis: even short courses of broad-spectrum antibiotics reduce microbial diversity, deplete commensal *Lactobacillus* and *Bifidobacterium* spp. and favor opportunistic colonization (*Gardnerella vaginalis*, *Candida albicans* or *Clostridium difficile*) [16,84]. In the female genital tract, these changes increase susceptibility to BV, recurrent vulvovaginal candidiasis and post-antibiotic dysbiosis [16]. Hormonal therapies (oral contraceptives and hormone replacement therapy) similarly modulate estrogen-dependent microbial communities by altering vaginal pH and glycogen availability [16,29]. Estrogen promotes glycogen deposition in the vaginal epithelium, supporting *Lactobacillus* dominance and lactic acid production, whereas hypoestrogenic states—physiological (menopause) or iatrogenic (GnRH analogs)—are associated with reduced *Lactobacillus*, elevated vaginal pH and increased anaerobic or pathogenic taxa [94]. Long-term hormonal contraceptive use may also influence gut microbiota composition, bile acid metabolism and systemic inflammation. In men, antibiotic exposure or androgen-suppressive therapies may similarly alter the seminal microbiota, with potential implication for fertility [95].

Clinical interest has therefore shifted toward microbiota modulation rather than eradication. Probiotics, live microorganisms, represent the most clinically advanced approach, with randomized trials and meta-analyses supporting their adjunctive use in preventing BV recurrence and restoring *Lactobacillus*-dominated eubiosis [96,97]. Intravaginal *L. crispatus* CTV-05 (LACTIN-V) following antibiotic therapy significantly reduces BV recurrence and promotes long-term colonization [65]. These interventions are increasingly considered in routine practice for recurrent BV, although their role in infertility, endometriosis, or gynecologic malignancies remains investigational.

Prebiotics, nondigestible substrates such as inulin, fructooligosaccharides (FOS), and galactooligosaccharides (GOS), selectively stimulate beneficial gut bacteria and may indirectly support genital tract homeostasis through immune modulation, especially after antibiotic or hormonal disruption [12]. However, clinical data in gynecologic populations remain limited.

Postbiotics, bioactive microbial metabolites, such as SCFA and cell wall components, are emerging as safer alternatives in immunocompromised patients or during pregnancy, exerting anti-inflammatory and barrier-enhancing effects and possibly improving endometrial receptivity via the gut–reproductive axis, although evidence is currently preclinical or early-phase [12,98].

Combined or sequential approaches (prebiotic plus probiotic or probiotic plus postbiotic) may enhance efficacy by providing both metabolic substrates and functional modulation. Next-generation probiotics, such as *Lactobacillus crispatus* CTV-05 or *L. rhamnosus* GR-1 and *L. reuteri* RC-14, administered orally or intravaginally, have demonstrated benefit in improving vaginal microbiota composition and reducing BV recurrence, particularly in women with recurrent dysbiosis or implantation failure [97].

Finally, fecal microbiota transplantation (FMT), although established for recurrent Clostridioides difficile infection, remains experimental in gynecological settings. Preliminary data suggest potential benefit in restoring intestinal eubiosis in women with severe dysbiosis and recurrent genital infections, but its application in reproductive medicine is not yet supported by controlled clinical trials [99].

Overall, microbiota modulation represents a promising adjunctive strategy in gynecological and obstetric care, but applications beyond recurrent BV should be considered investigational. Future research should prioritize randomized trials and personalized microbiome-based interventions integrating genomic, metabolomic, and hormonal profiling [100].

## 8. Microbiota and Gynecologic Surgery

Gynecologic surgeries induce significant alterations in both genital and intestinal microbiota through mechanical disruption, antibiotic exposure, endocrine changes, and surgical stress. Instrumentation of the cervico-vaginal canal and uterine cavity may facilitate microbial translocation, allowing taxa typically confined to the lower genital tract (*Gardnerella*, *Prevotella*, *Atopobium*, *Streptococcus*, *Enterococcus*, *Escherichia*, *Shigella*) to access deeper tissues or the bloodstream, transiently amplifying inflammation. Perioperative factors including antibiotic prophylaxis, surgical stress, bowel preparation, and anesthesia further reduce microbial diversity and favor opportunistic colonization [101]. Postoperative hormonal changes further influence microbial composition. A case–control study in women undergoing transabdominal hysterectomy for uterine fibroids showed increased postoperative follicle-stimulating hormone (FSH) levels, reduced estradiol (E2) and anti-Müllerian hormone (AMH), indicating altered ovarian function despite ovarian preservation [102]. These hormonal changes were associated with gut dysbiosis, characterized by increased *Proteobacteria* and *Firmicutes* and reduced *Bacteroidetes* [101,102].

Cohort studies suggest that surgery produces both acute (transient imbalance with increased *Escherichia*/*Shigella* and *Enterococcus* and reduced *Lactobacillus* dominance) and long-term microbial remodeling, with early postoperative dysbiosis followed by partial recovery of beneficial taxa [103,104,105]. These shifts may influence postoperative recovery, infection risk, and long-term inflammatory and metabolic homeostasis [41].

## 9. Conclusions

Accumulating evidence indicates that alterations of the vaginal, endometrial, and gut microbiota are consistently associated with a broad spectrum of gynecological and obstetric conditions (Table 1). Although most data remain observational, dysbiosis is linked to inflammatory activation, hormonal imbalance, and impaired reproductive function, underscoring the systemic interplay between microbial, endocrine, and immune pathways within the gut-reproductive axis. Methodological heterogeneity, limited sample sizes, and challenges related to low-biomass sampling currently limit causal inference and clinical translation. At present, microbiota profiling should be considered investigational, and microbiome-based interventions adjunctive. Well-designed longitudinal and interventional studies integrating multi-omics approaches are essential to clarify causality and support the development of personalized microbiota-guided strategies in women’s reproductive health.

## Figures and Tables

**Table 1:** Comprehensive summary table of microbiota alterations associated with major gynecological and obstetric conditions. Bacterial vaginosis (BV); sexually transmitted infections (STIs); pelvic inflammatory disease (PID).

Condition	Site	Typical Dysbiosis	Taxa	Key Mechanisms	Level of Evidence
BV	Vagina	Loss of *Lactobacillus* dominance; high microbial diversity	*Gardnerella vaginalis*, *Atopobium vaginae*, *Prevotella*, *Mobiluncus*, *Sneathia*, BVAB1–3	Increased vaginal pH; biofilm formation; epithelial barrier disruption; production of sialidases, proteases, SCFA; local inflammation	High (meta-analyses, RCTs for treatment)
STIs	Vagina , Cervix	*Lactobacillus* depletion; anaerobe enrichment	*Gardnerella*, *Prevotella*, *Sneathia*, reduced *L. crispatus*	Impaired mucosal barrier; increased pH; enhanced pathogen adhesion; reduced innate immune protection	High (epidemiological + mechanistic)
PID	Upper Genital Tract	BV-like vaginal microbiota with ascending infection	*Gardnerella*, *Atopobium*, *Prevotella*, *Sneathia*, *Mobiluncus*	Cervical mucus degradation; microbial ascent; TLR-mediated inflammation; cytokine release (IL-6, IL-8, TNF-α); tubal damage	Moderate (observational, molecular studies)
Infertility/implantation failure	Vagina, Endometrium	Reduced *Lactobacillus* dominance; increased diversity	*Gardnerella*, *Streptococcus*, *Prevotella*, *Atopobium*	Endometrial inflammation; impaired receptivity; LPS-induced immune activation; altered cytokine milieu	Moderate (ART cohorts)
Male factor infertility	Semen	High diversity; enrichment of anaerobes	*Prevotella*, *Finegoldia*, *Campylobacter*, *Porphyromonas*	Seminal inflammation; oxidative stress; sperm motility and DNA damage; partner microbial exchange	Low–moderate (descriptive metagenomics)
Endometriosis	Gut, Endometrium	Gut dysbiosis; reduced beneficial taxa	Reduced *Lactobacillus*, *Bifidobacterium*; increased *Proteobacteria*	Increased gut permeability; systemic inflammation; estrogen recirculation (β-glucuronidase activity); immune dysregulation	Moderate (associative, mechanistic hypotheses)
Chronic endometritis	Endometrium	Non-Lactobacillus-dominant low-biomass microbiota	*Gardnerella*, *Streptococcus*, *Enterococcus*, *Escherichia*	Persistent endometrial inflammation; altered implantation window; immune cell activation	Moderate (histologic + sequencing studies)
Preterm birth	Vagina, Cervix, Uterus	BV-like dysbiosis	*Gardnerella*, *Atopobium*, *Ureaplasma*, *Prevotella*	Ascending infection; inflammatory activation of fetal membranes; cytokine cascade; sterile inflammation	High (meta-analyses)
Recurrent miscarriage	Vagina, Endometrium	Reduced Lactobacillus dominance	*Gardnerella*, *Atopobium*, *Streptococcus*	Impaired implantation; abnormal placentation; inflammatory signaling	Moderate (cohort studies)
Gynecologic malignancies	Vagina, Uterus, Gut	Increased diversity; loss of protective taxa	Reduced *Lactobacillus*; increased anaerobes	Chronic inflammation; immune evasion; altered estrogen metabolism; tumor microenvironment modulation	Low–moderate (associative)
Post-gynecologic surgery states	Vagina, Gut	Transient dysbiosis; loss of commensals	Increased *Enterococcus*, *Escherichia*/*Shigella*; reduced *Lactobacillus*	Surgical disruption; antibiotics; hormonal changes; inflammation	Moderate (cohort studies)

## Data Availability

No new data were created.
